# The Denitrification Characteristics and Microbial Community in the Cathode of an MFC with Aerobic Denitrification at High Temperatures

**DOI:** 10.3389/fmicb.2017.00009

**Published:** 2017-01-19

**Authors:** Jianqiang Zhao, Jinna Wu, Xiaoling Li, Sha Wang, Bo Hu, Xiaoqian Ding

**Affiliations:** ^1^School of Environmental Science and Engineering, Chang'an UniversityXi'an, China; ^2^Key Laboratory of Subsurface Hydrology and Ecological Effect in Arid Region of Ministry of EducationXi'an, China; ^3^School of Civil Engineering, Chang'an UniversityXi'an, China

**Keywords:** aerobic denitrifying bacteria, dissolved oxygen, microbial fuel cell, predominant species, simultaneous nitrification and denitrification

## Abstract

Microbial fuel cells (MFCs) have attracted much attention due to their ability to generate electricity while treating wastewater. The performance of a double-chamber MFC with simultaneous nitrification and denitrification (SND) in the cathode for treating synthetic high concentration ammonia wastewater was investigated at different dissolved oxygen (DO) concentrations and high temperatures. The results showed that electrode denitrification and traditional heterotrophic denitrification co-existed in the cathode chamber. Electrode denitrification by aerobic denitrification bacterium (ADB) is beneficial for achieving a higher voltage of the MFC at high DO concentrations (3.0–4.2 mg/L), while traditional heterotrophic denitrification is conducive to higher total nitrogen (TN) removal at low DO (0.5–1.0 mg/L) concentrations. Under high DO conditions, the nitrous oxide production and TN removal efficiency were higher with a 50 Ω external resistance than with a 100 Ω resistance, which demonstrated that electrode denitrification by ADB occurred in the cathode of the MFC. Sufficient electrons were inferred to be provided by the electrode to allow ADB survival at low carbon:nitrogen ratios (≤0.3). Polymerase chain reaction-denaturing gradient gel electrophoresis (PCR-DGGE) results showed that increasing the DO resulted in a change of the predominant species from thermophilic autotrophic nitrifiers and facultative heterotrophic denitrifiers at low DO concentrations to thermophilic ADB at high DO concentrations. The predominant phylum changed from *Firmicutes* to *Proteobacteria*, and the predominant class changed from *Bacilli* to *Alpha, Beta*, and *Gamma Proteobacteria*.

## Introduction

Microbial fuel cells (MFCs) have gained widespread attention as an innovative wastewater treatment and energy recovery technology that combines sewage purification and electricity production (Janicek et al., 2014; Li et al., 2014). Recent studies have shown that nitrate and nitrite can be removed from wastewater as electron acceptors in the cathode of an MFC through electrochemical reduction or autotrophic denitrification (Zhang and Angelidaki, 2012). Several developments of nitrogen removal with MFCs have been achieved with various designs and configurations (He et al., 2009; Virdis et al., 2010; Zhang and Angelidaki, 2013). In the studies of Bernardino Virdis et al., the cathodic process with *in situ* nitrification through specific aeration attained simultaneous nitrification and denitrification (SND) in one half-cell (Virdis et al., 2010). Although, nitrogen recovery with MFCs through NH_3_ stripping has been successfully developed to simultaneously produce energy and recover ammonium (Kuntke et al., 2012; Zhang and Angelidaki, 2015), SND in cathode of MFCs and its some new biochemical mechanisms still remain valuable to explore. Studies of simultaneous phenol removal, nitrification and denitrification using MFCs have indicated that phenol-degrading bacteria, nitrifiers, and denitrifiers in the aerobic cathode chamber are responsible for phenol oxidation, aerobic nitrification and aerobic denitrification, respectively (Feng et al., 2015). The impact of dissolved oxygen (DO) on the SND process in the cathode of an MFC has also been investigated comprehensively (Virdis et al., 2010). Because the bacteria may evolve during long-term operation, the impact of DO on the performance of cathode denitrification is different from that over shorttime periods. The SND mechanism in the aerobic cathode chamber is complex and remains unclear.

In the traditional theory of biological nitrogen removal, ammonia is first oxidized to nitrate by autotrophic nitrifiers, and the nitrate is then reduced to nitrogen by heterotrophic denitrifiers (Robertson and Kuenen, 1984). Based on the different growth conditions of nitrifiers and denitrifiers, the traditional theory of biological nitrogen removal makes a strict distinction between the nitrification and denitrification processes. The former is carried out under aerobic conditions, while the latter requires anaerobic conditions. Therefore, it is impossible for the two reactions to occur simultaneously in the same reactor. However, the discovery of heterotrophic nitrifiers and aerobic denitrifiers has made it possible for nitrification and denitrification to occur simultaneously (Huang et al., 2013; Li et al., 2015). Heterotrophic nitrifying bacteria can produce hydroxylamine, nitrite and nitrate by nitrification using organic carbon as a source for growth, and most of these bacteria can also directly convert nitrifying products to nitrogen gas through the process of aerobic denitrification (Papen and Von Berg, 1998). Aerobic denitrification bacterium (ADB) can use aerobic denitrifying enzymes for denitrification under aerobic conditions (Robertson et al., 1988; Bell and Ferguson, 1991).

In the 1980s, Robertson and Kuenen (1984) isolated the aerobic denitrifiers *Thiosphaera pantotropha, Pseudomonas* spp. and *Alcaligenes faecalis* for the first time and reported the existence of the aerobic denitrifying enzyme system (Robertson et al., 1988). They also confirmed that the growth rate of *Paracoccus denitrificans* will be higher in the presence of O_2_ and ${\text{NO}}_{3}^{-}$. Bell and Ferguson (1991) demonstrated that aerobic denitrifying enzymes were more active in the presence of O_2_, and Meiberg et al. (Ferguson, 1994) reported that denitrification could be carried out by *Hyphomicrobium X* under aerobic conditions. Many studies have proved the existence of ADB (Chen et al., 2003; Kim et al., 2005) and found that some denitrifiers survive under high O_2_ concentration conditions (Takaya et al., 2003). Certain groups of bacteria, such as *Bacillus, P. putida, P. stutzeri, Hydrogenophaga*, and *Achromobacter*, have been shown to have heterotrophic nitrification and aerobic denitrification abilities and to convert ammonium to nitrogen aerobically in the cathode chamber of an MFC (Feng et al., 2015). Nevertheless, few attempts have been made to attain SND at high temperatures. Because some wastewater, similar to sludge digestion solutions and effluents of anaerobic reactors that treat landfill leachate, contains high concentrations of ammonia at high temperatures, studies of SND and the performance of ADB in the cathode of an MFC at high temperatures are important.

This study investigated the performance of a double-chamber MFC with SND in the cathode at fluctuating high temperatures (36–48°C). Synthetic wastewater that contained organics and high concentrations of ammonia was fed into the anode chamber and then turned into the cathode chamber. The denitrification characteristics were studied by comparing scenarios with two ranges of DO concentrations (0.5–1.0 and 3.0–4.2 mg/L) and scenarios with two external resistances (50 and 100 Ω) at high DO concentrations. The microbial communities at the two DO concentrations in the cathode of the MFC were identified with polymerase chain reaction-denaturing gradient gel electrophoresis (PCR-DGGE) to explore the evolution of the dominant bacteria.

## Materials and methods

### Experimental set-up

**Figure 1:**
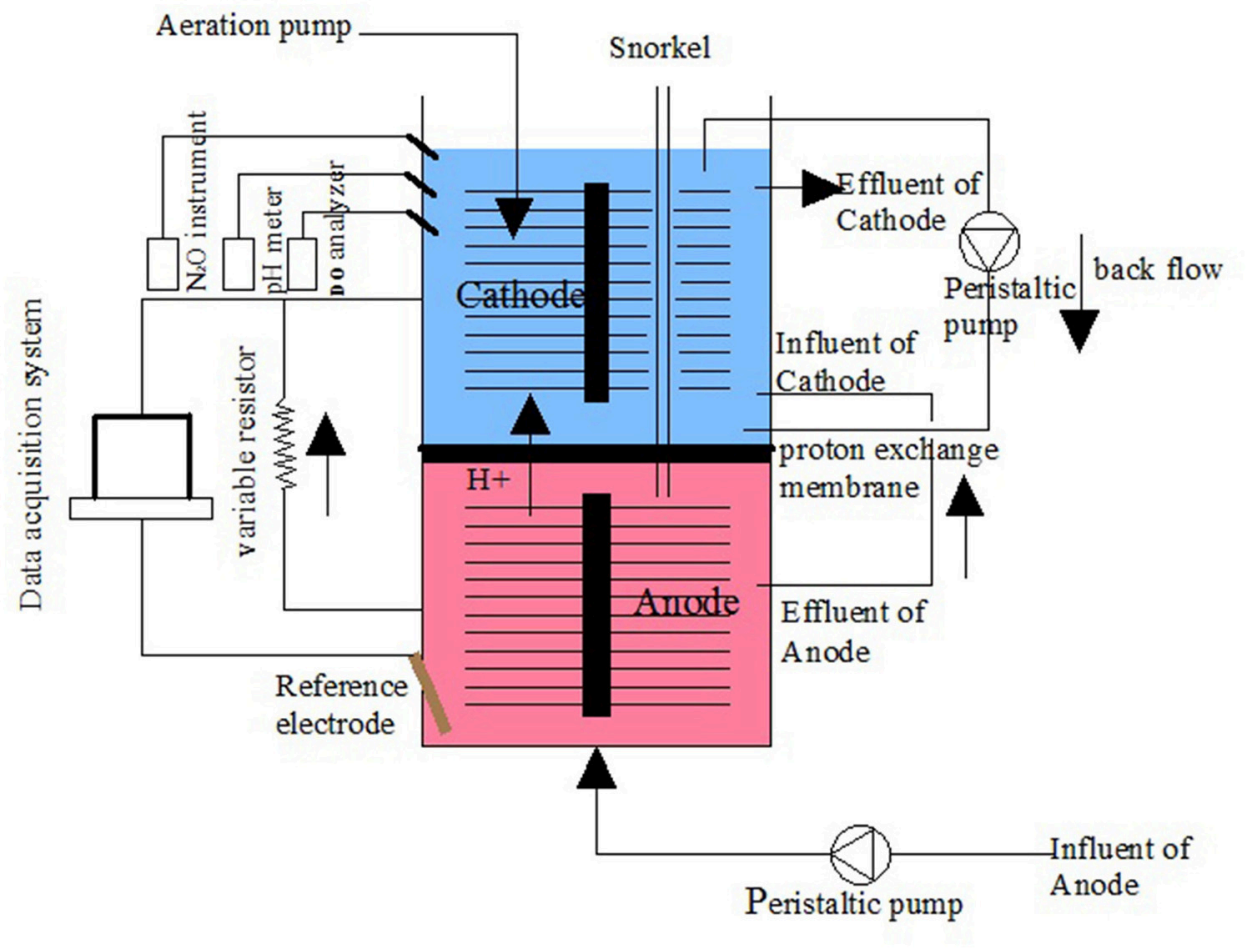
**Schematic of the double chamber MFC**.

The MFC device was constructed with cathode and anode chambers. The anode and cathode chambers were both made of organic glass tube 8 cm high and 9 cm in diameter and had an effective volume of 0.452 L (Figure 1). Each chamber used a carbon brush as the electrode. The two chambers were separated by a proton exchange membrane (Nafion 117) and placed in a water bath. The temperature was initially set to 31 ± 1°C and then changed to a dynamic temperature (36–48°C) later in the operation. The cathode and anode chambers were connected with a manual variable resistor (0–9999 Ω) to close the circuit. The cathode chamber was exposed to air, and blast aeration was used. The influent was injected into the anode chamber using a peristaltic pump (YZ1515X, Lange), and the effluent from the anode was fed into the cathode chamber.

### Influent component

The influent for the MFC reactor was an artificially simulated high strength ammonia sludge digestion solution with components of 0.38 g/L CH_3_COONa, 2.708 g/L NH_4_HCO_3_, 0.33 g/L KH_2_PO_4_, 1 g/L K_2_HPO_4_·3H_2_O, 1 g/L KCl, 1.5 g/L NaHCO_3_, 0.016 g/L CaCl_2_, and 1 ml/L trace nutrient solution. CH_3_COONa and NH_4_HCO_3_ were added to maintain the chemical oxygen demand (COD) and the ammonia nitrogen concentrations in the influent at 300 and 480 mg/L, respectively.

### Start-up and operation

The anode and cathode chambers of the MFC were inoculated with aerobic sludge from the aeration tank of the Fourth Wastewater Treatment Plant in Xi'an, China. Before operating the MFC, the anode and cathode were soaked in the inoculation sludge for 24 h. Once the reactor was filled with synthetic wastewater, the MFC entered the stage of static culture without the influent while keeping the cathode aerated and the inner circuit open. The concentrations of DO, ${\text{NO}}_{3}^{-}$-N, ${\text{NO}}_{2}^{-}$-N, and ${\text{NH}}_{4}^{+}$-N and the pH of the cathode electrolyte were measured daily. After more than half of the original ${\text{NH}}_{4}^{+}$-N was converted to ${\text{NO}}_{2}^{-}$-N in the cathode electrolyte, the aeration mode was changed from continuous to intermittent (2 h aeration and 2 h static), and the wastewater was pumped continuously into the anode chamber with hydraulic retention times (HRTs) of 10.4 h for both the cathode and the anode chambers. After a period of continuous operation, stable partial nitrification was obtained in the cathode chamber of the MFC. An external resistance of 100 Ω was then connected, following which the operation of MFC with SND started.

### Analytical method

The anode potential was monitored with a saturated calomel electrode (SCE, +0.242 V standard hydrogen electrode; Type 232, Leici Instrument Factory, Shanghai, China). ${\text{NO}}_{3}^{-}$-N, ${\text{NO}}_{2}^{-}$-N and ${\text{NH}}_{4}^{+}$-N were measured according to Standard Methods for the Examination of Water and Wastewater (Clesceri et al., 1998). DO was determined using a Hach-HQ30d DO analyzer (HACH, America). The voltage and anode potential were monitored and recorded using a PCI1717 voltage collector (Yanhua Company, Shenzhen, China). An N_2_O microsensor (Unisense, Denmark) was used for the N_2_O analysis.

Samples from the biofilm of the cathode were collected on day 27 and day 83 to investigate the microbial community with DGGE, and DNA was extracted using a fast DNA spin kit (SK8233) for soil according to the manufacturer's instructions. The bacterial 16S rRNA genes were amplified by PCR with the universal primers F357-GC (5′-CGCCCGCCGCGCCCCGCGCCCGGCCCGCCGCCCCGCCCCCCTACGGGAGGCAGCAG-3′) and R518 (5′-ATTACCGCGGCTGCTGG-3′). A polyacrylamide gel (8%) with a 30–60% denaturing gradient was used to separate the PCR products (7 mol L-1 urea and 40% formamide comprising 100% denaturant), which were analyzed using DGGE technology and washed with ultrapure water to flush the gel and dye. Eight representative DGGE strips were selected by a clean scalpel and transferred to a 1.5 mL centrifuge tube. The target DNA fragments were then excised and reamplified using the primer sets F357 (5′-CCTACGGGAGGCAGCAG-3′) and R518 (5′-ATTACCGCGGCTGCTGG-3′), and the obtained sequence was matched with the Seqmatch database for sequence alignment. The homology information of each strip was obtained by Sangon Biotech Co., Ltd. (Shanghai, China).

## Results and discussion

### Performance of the MFC

**Figure 2:**
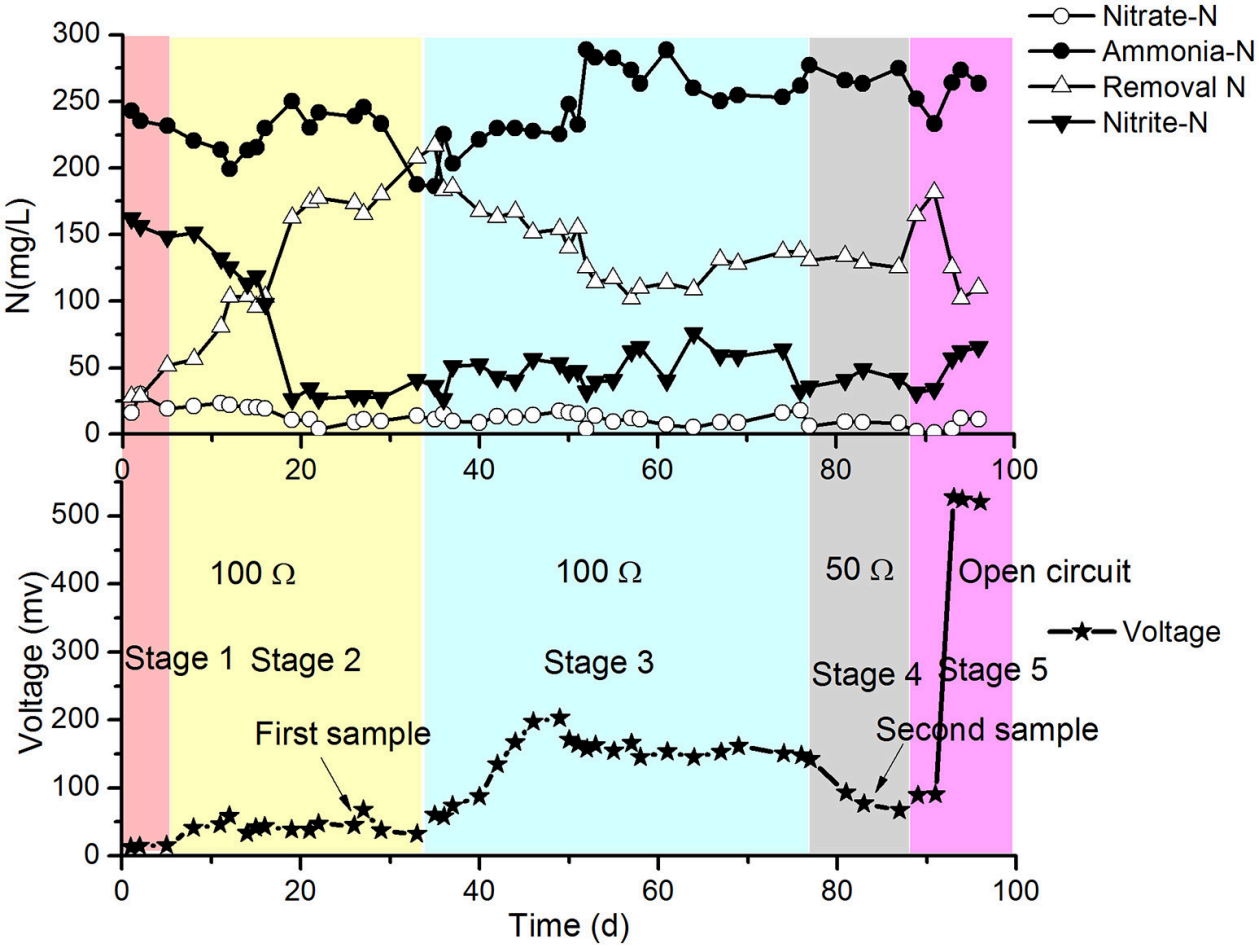
**Profiles of ${\text{NH}}_{4}^{+}$-N, ${\text{NO}}_{2}^{-}$-N and ${\text{NO}}_{3}^{-}$-N in the effluent of the cathode chamber and voltages of the MFC**.

The results of the continuous operation test are shown in Figure 2.

In stage 1 (first 5 days of the test), the MFC with SND was operated at 31 ± 1°C, 100 Ω resistance and intermittent aeration (2 h aeration and 2 h static), and the DO of the catholyte was 0.5–1.0 mg/L. The concentrations of ${\text{NH}}_{4}^{+}$-N and ${\text{NO}}_{2}^{-}$-N decreased with a gradual increase in the release voltage of the MFC. However, the removal of total nitrogen (TN) was much greater than that with the electrical current of the MFC. This finding implies that the traditional heterotrophic microbial denitrification with COD was more efficient at removing nitrogen than the electrode denitrification in the cathode chamber. The moderate temperature (31 ± 1°C) and low DO (0.5–1.0 mg/L) in the cathode may be beneficial for SND with COD still present in the anode effluent.

In stage 2 (days 6–34), the temperature of the reactor increased and fluctuated over the range of 36–48°C. The removal of TN began to increase sharply, and the concentration of ${\text{NO}}_{2}^{-}$-N in the cathode effluent began to decrease correspondingly from the 6th day, while the voltage of the MFC increased slightly and then stabilized. This might have been due to the high temperature in the cathode (36–48°C), which was harmful for the growth of normal ammonia oxidizing bacteria (AOB). Because the suitable range of temperatures for AOB metabolism is 20–30°C, the nitrification rate decreased, and the ${\text{NO}}_{2}^{-}$-N concentration decreased to approximately 30 mg/L. The increase in the TN removal was mainly caused by the volatilization of ammonium at high temperature. The TN removal from the denitrification in the electrode during stage 2 was lower and similar to that at the end of stage 1 (not greater than 7 mg/L·d based on the MFC voltage). The biofilm was sampled, and the bacteria were identified with PCR-DGGE. The predominant species was found to be *Ureibacillus thermosphaericus* of the genus *Ureibacillus*, which grows at temperatures of 37° to 60°C (Fortina et al., 2001).

In stage 3 (days 35–77), the reactor was set to continuous aeration instead of intermittent aeration. As a result, the DO increased to 3 ± 0.6 mg/L. The amount of TN removed per day began to decrease, but the voltage continued to increase. This might have occurred because the heterotrophic denitrification with COD as the electron donor was inhibited by the increase of DO, and the partial oxygen accepted electrons from the electrode. In stage 3, the release voltage initially increased, then dropped and finally steadied at approximately 100 mV, which was much higher than that in stage 2. The following strain sampling clearly demonstrated that the predominant species changed to aerobic denitrifiers (a detailed analysis is provided later). The curves in Figure 2 suggest that the aerobic denitrifiers might have replaced the anaerobic denitrifiers in the latter phase of stage 3. The aerobic denitrifiers appeared to be much more receptive to the electrons from the electrode than the anaerobic denitrifiers, which was determined by comparing the voltage of stage 2 with that of stage 3, in which the effects of electron acceptance by oxygen was taken into account. In contrast, TN removal by anaerobic denitrifiers was much greater than that by aerobic denitrifiers. The heterotrophic denitrification with COD might have mainly caused TN removal, excluding the effect of volatilization in the cathode. Depending on the voltage, the TN removal by electrode denitrification was 8.5–9.0 mg/(L·d), which represented only a small part of the TN removal in stage 3.

The conditions in stage 4 (days 78–88) remained the same as those in stage 3 except for the change in the resistance of the MFC from 100 to 50 Ω. The results showed that the concentrations of ${\text{NH}}_{4}^{+}$-N and ${\text{NO}}_{2}^{-}$-N in the cathode effluent decreased, and the amount of TN removed increased in the latter part of this stage (Figure 2). The voltages were greater than those in stage 3, but the TN removal by electrode denitrification was 13.5–14.6 mg/(L·d), which was greater than that in stage 3.

### Electricity production and nitrogen removal performance under high DO conditions

**Figure 3:**
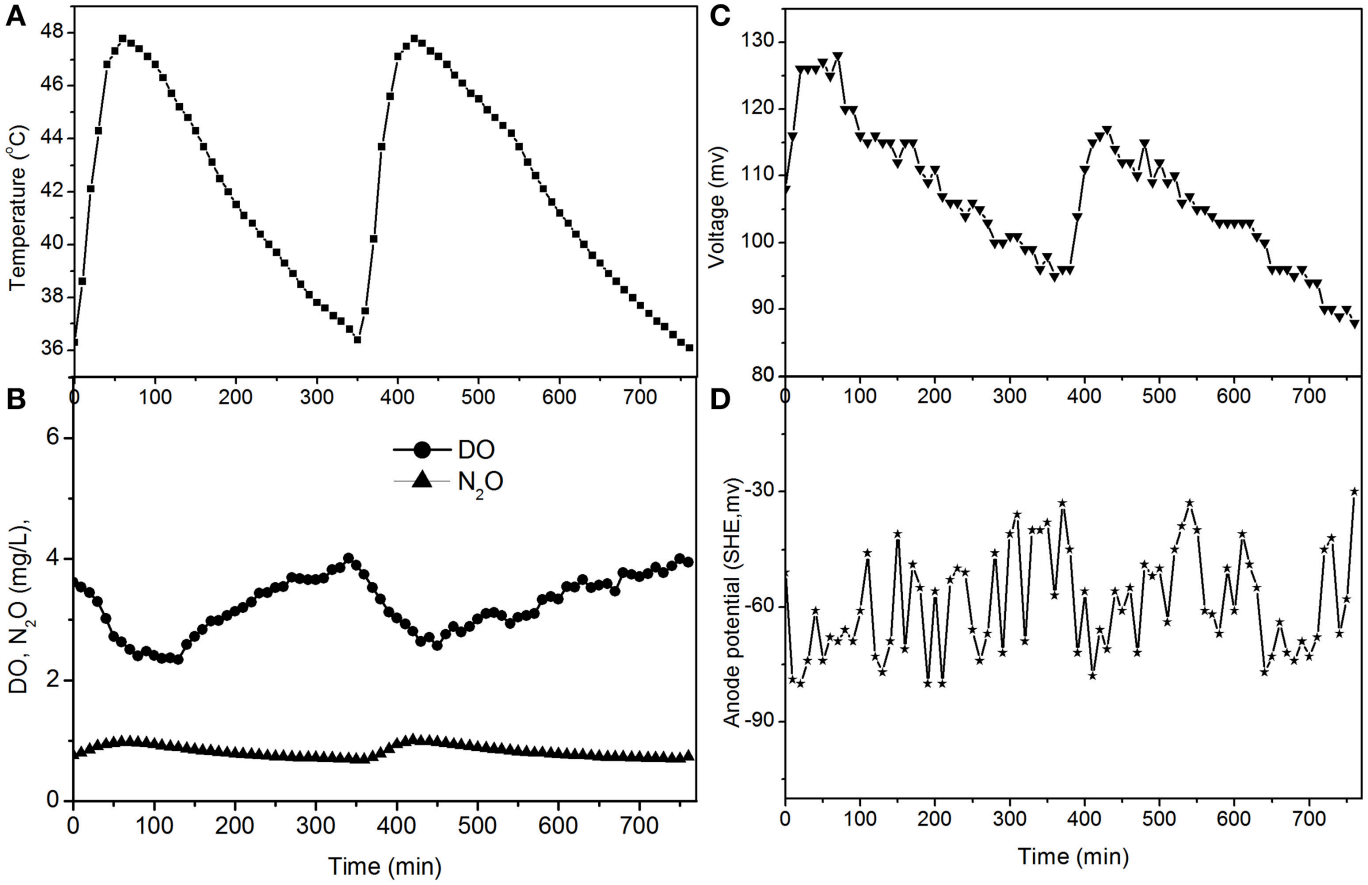
**Profiles of temperature, DO, N_2_O, voltage, and anode potential of the MFC with 100 Ω external resistance at temperatures of 36–48°C (day 75 in Figure 2). (A)** Temperature profile; **(B)** DO and N_2_O profiles; **(C)** voltage profile; **(D)** anode potential profile.

**Figure 4:**
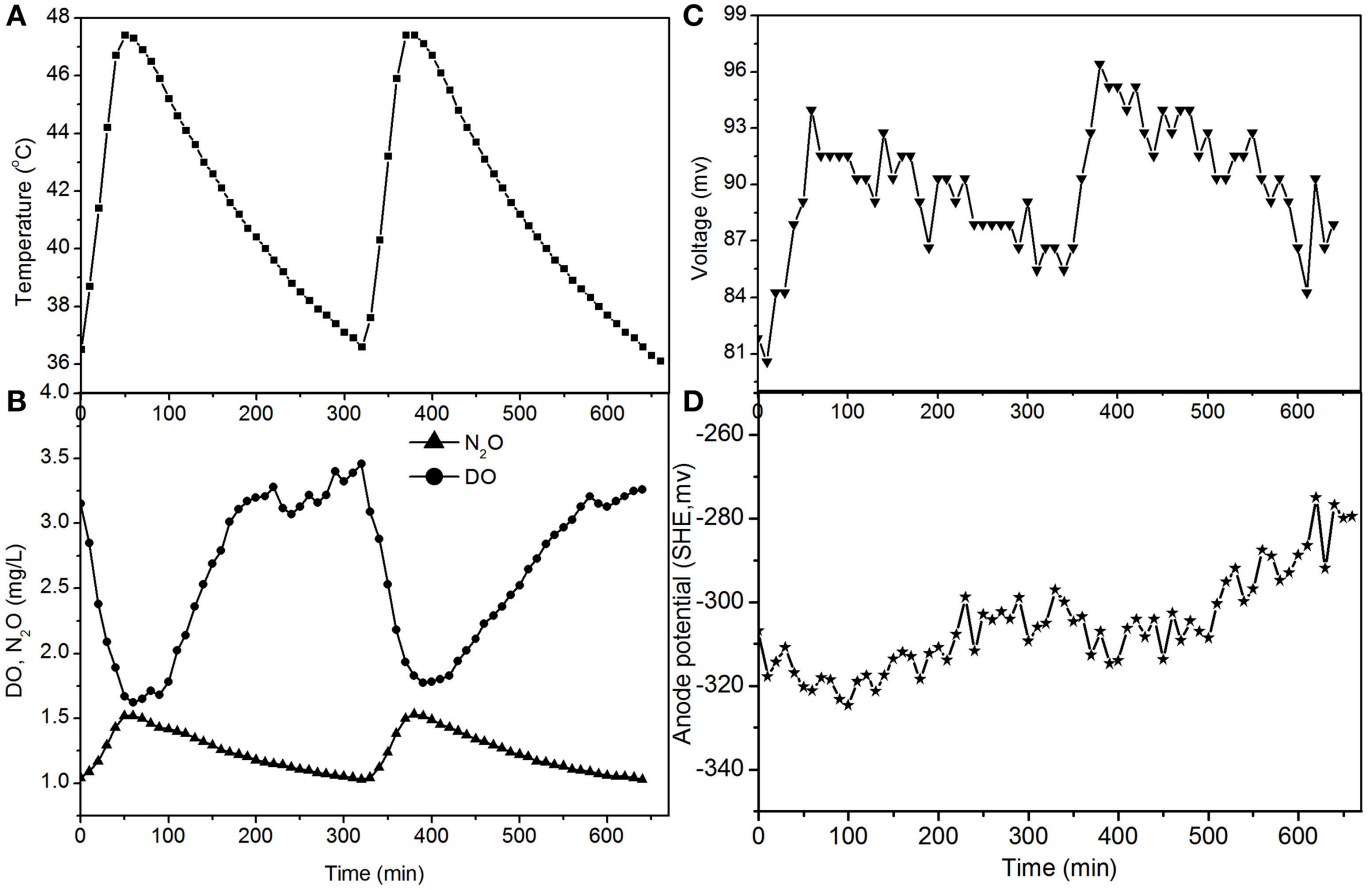
**Profiles of temperature, DO, N_2_O, voltage, and anode potential of the MFC with 50 Ω external resistance at temperatures of 36–48°C (day 82 in Figure 2). (A)** Temperature profile; **(B)** DO and N_2_O profiles; **(C)** voltage profile; **(D)** anode potential profile.

**Figure 5:**
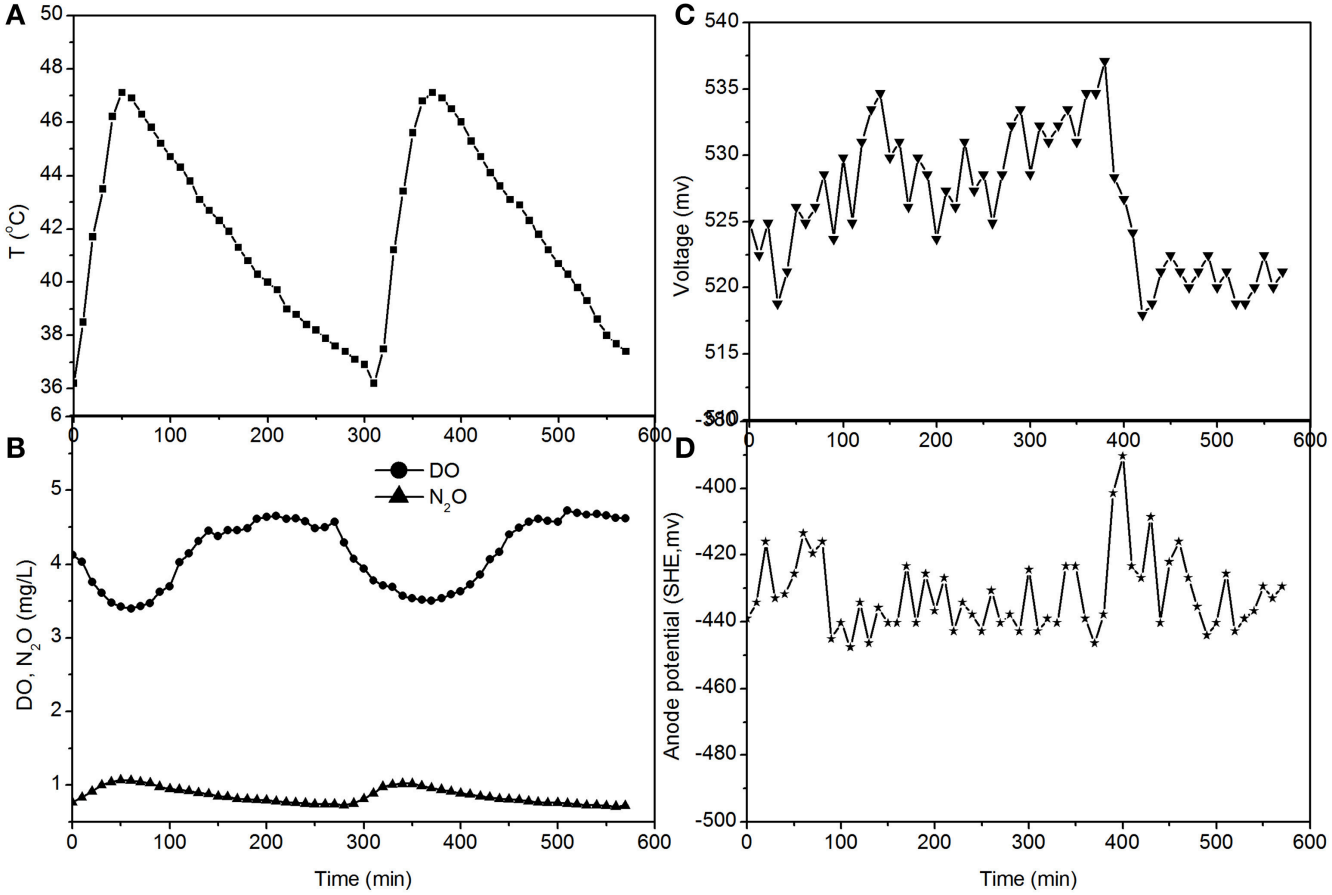
**Profiles of temperature, DO, N_2_O, voltage, and anode potential of the MFC in open circuit at temperatures of 36–48°C (day 97 in Figure 2). (A)** Temperature profile; **(B)** DO and N_2_O profiles; **(C)** voltage profile; **(D)** anode potential profile.

The electricity production performance of the MFC under high DO conditions and different external resistances was investigated as a case study (Figures 3–**5**, which correspond to days 75, 82, and 97 in Figure 2, respectively). The voltage was positively related to the increase in temperature under external resistances of 50 Ω and 100 Ω, while it was independent of the temperature under open circuit conditions. Regardless of whether the resistances were applied or an open circuit was used, the N_2_O emissions were always positively related to the increase in temperature, while the DO was always negatively related to the increase in temperature. The characteristics of denitrification and electricity production of the MFC can be evaluated using these factors.

The fluctuations in the concentrations of DO and N_2_O caused by the temperature (Figures 3, **5**) show that the DO fluctuation amplitude was approximately 1.2 mg/L, whereas the corresponding N_2_O fluctuation was approximately 0.4 mg/L in the open circuit. With a 100 Ω resistance, the DO and N_2_O fluctuation amplitudes were 1.5 mg/L and 0.3 mg/L, respectively. This demonstrated that the reduction in oxygen caused by the voltage fluctuation was approximately 0.3 mg O_2_/L, and no N_2_O was produced by electrode denitrification. The concentrations of N_2_O were similar in both scenarios (0.7–1.1 mg/L), whereas the concentrations of DO with a 100 Ω resistance were 0.7–1.0 mg/L, which was lower than that in the open circuit (Figures 3, **5**). This indicated that the decrease in DO was dependent on the electrode reaction, while the production of N_2_O was independent of the electrode reaction. Using Coulomb's law, with a 100 Ω resistance, the electrode reduction rate of oxygen was calculated to be 1.6–2.3 mg O_2_/(L·d), whereas the rate of electrode reduction of nitrite to nitrogen gas was 8.5–9.0 mg N/(L·d) with no production of N_2_O.

The fluctuations in the concentrations of DO and N_2_O caused by temperature (Figures 4, 5) show that the decrease of DO and the increase of N_2_O were both dependent on the electrode reaction. Using Coulomb's law, with a 50 Ω resistance, the electrode reduction rate of oxygen was calculated to be 2.8–4.4 mg O_2_/(L·d), whereas the rate of electrode denitrification was 13.5–14.6 mg N/(L·d), in which approximately 10% of the nitrogen removed was converted to N_2_O and 90% was converted to nitrogen gas.

The N_2_O production and TN removal efficiencies with a 50 Ω external resistance were higher than those with a 100 Ω resistance, which illustrates that electrode denitrification occurred in the cathode of the MFC. These analyses indicate that both oxygen and nitrite can obtain electrons simultaneously from the electrode in the cathode of the MFC.

### Performance of the microbial community at low DO and high temperature

**Figure 6:**
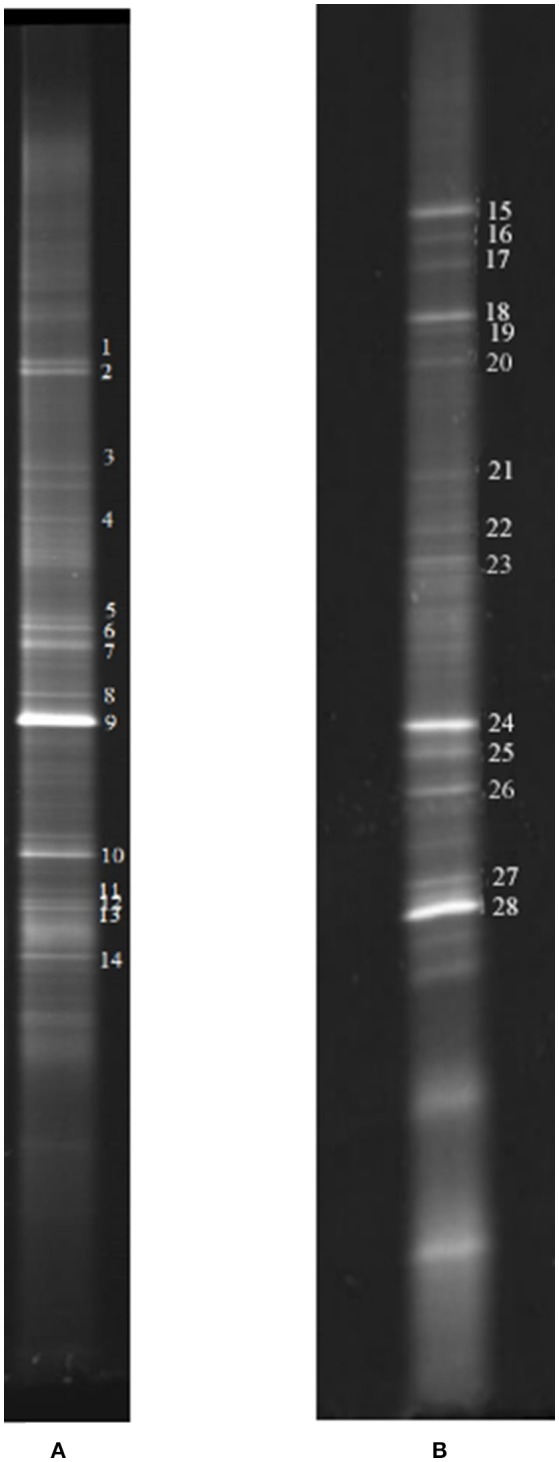
**Representative DGGE strips of samples. (A)** Sampling at low DO levels (first sampling); **(B)** sampling at high DO levels (second sampling).

Biofilm samples were collected from the cathode chamber of the MFC on day 27 in stage 2 (Figure 2) and day 83 in stage 4 (Figure 2). The representative DGGE strips are shown in Figure 6. The closest species and classification of each representative band in the DGGE profile were deposited in the GenBank database (Tables 1, 2). Sequences from DGGE bands are shown in Supplementary Material.

**Table 1:** **Identification of DGGE bands under low DO conditions (the first sampling; Figure 6A)**.

**Band**	**Taxon**	**Similarity (%)**	**Accession**	**Phylum/Genus**	
2	*Ureibacillus thermosphaericus*	91.2	AF403017	*Firmicutes/Ureibacillus*	Aerobic, thermophilic, grows at 37–60°C, optimum at 50–60°C, negative for nitrate reduction, anaerobic growth, acid production from glucose (Fortina et al., 2001).
4	As above	100	As above	As above	
5	As above	94.6	As above	As above	
8	As above	92.5	As above	As above	
9	As above	97.3	As above	As above	
7	As above	95.7	As above	As above	
10	*Bacillus* sp. R-7413	100	AY422985	*Firmicutes/Bacillus*	Heterotrophic, anaerobic, nitrate reduction, optimum at 70°C (Kim et al., 1998)
13	*Anoxybacillus kaynarcensis*	97.3	EU926955	*Firmicutes/Anoxybacillus*	Heterotrophic, aerobic, can reduce nitrate to nitrite, grows from approximately 35 to 70°C, optimum at 60°C (Inan et al., 2013).
3	*Geobacillus toebii*	92.4	EU428777	*Firmicutes*/*Geobacillus*	Heterotrophic, aerobic, nitrate and nitrite reduction positive, grows from 55 to 75°C, optimum at 68°C (Poli et al., 2006).
	*Anoxybacillus calidus*	*100*	FJ430012	*Firmicutes/Anoxybacillus*	Facultatively anaerobic, heterotrophic, N_2_ gas produced from nitrate, 35–70°C, optimum at 55°C (Cihan et al., 2014).
1	*Uncultured planctomycete*	100	GQ35	*Ignavibacteriae/Ignavibacterium*	Refers to a strain of *Ignavibacterium album* Mat9-16T, strictly anaerobic, heterotrophic, grows at 30–55°C, optimum at 45°C (Iino et al., 2010).
6	*Uncultured Chloroflexi bacterium*	83.4	JN825481	*Chloroflexi/Unclassified Anaerolineaceae*	Refers to a strain of *Anaerolinea thermolimosa* IMO-1T, strictly anaerobic, heterotrophic bacteria, cannot utilize nitrate as electron acceptors, grows at 42–55°C optimum at 50°C (Yamada et al., 2006).
14	*Cucumibacter marinus (T)*	96.8	EF211830	*Proteobacteria/Cucumibacter*	Heterotrophic aerobic bacteria, cannot reduce nitrate to nitrite, grows at 15–40°C, optimum at 30–35°C (Hwang and Cho, 2008).

**Table 2:** **Identification of DGGE bands under high DO conditions (second sampling; Figure 6B)**.

**Band**	**Taxon**	**Similarity (%)**	**Accession**	**Phylum/Genus**	**Properties**
15	*Aquamicrobium aestuarii*	100	GU199003	*Proteobacteria/Aquamicrobium*	Grows at 15–45°C, optimum at 30–35°C, can reduce nitrate to nitrite, strictly aerobic, heterotrophic bacteria (Jin et al., 2013).
25	*Brevundimonas diminuta*	98.1	X87274	*Proteobacteria/Brevundimonas*	*Brevundimonas* gen. nov, is aerobic, grows at 30–37°C, cannot reduce nitrate, 90% of the strains are autotrophic (Segers et al., 1994).
26	*Uncultured bacterium*	95.7	EF173342	*Proteobacteria/Altererythrobacter*	Refers to *Altererythrobacter epoxidivorans* JCS350T, cannot reduce nitrate, grows at 20–40°C, aerobic, optimum at 35°C, heterotrophic bacteria (Kwon et al., 2007).
18	*Pelomonas saccharophila* (T)	100	AB021407	*Proteobacteria/Pelomonas*	Grows at 4–40°C, optimum at 25–32°C, aerobic, able to fix nitrogen and show autotrophic growth with hydrogen but not photoautotrophic. Glucose and acetate are utilized as carbonsources for growth but negative for denitrification (Xie and Yokota, 2005).
27	*Brachymonas* sp. canine oral taxon 015	89.4	JN713175	*Proteobacteria/Brachymonas*	Refers to *Brachymonas denitrificans*, aerobic, denitrification positive, grows at 10–40°C, optimum at 30–35°C (Hiraishi et al., 1995).
	*Comamonas denitrificans*	87.8	AF233876	*Proteobacteria/Comamonas*	Grows at 20, 30, and 37°C, aerobic, heterotrophic, can reduce nitrate to nitrogen gas and contains cd1-type nitrite reductase (the only species in the genus *Comamonas* to do so) (Xing et al., 2010).
24	*Alishewanella* sp. N5	90.1	EU287929	*Proteobacteria/Alishewanella*	Refers to *Alishewanella aestuarii*, grows at 18–44°C, aerobic, optimum at 37°C, can reduce nitrate to nitrite and nitrogen gas, maltose is assimilated, heterotrophic bacteria (Roh et al., 2009).
28	*Acinetobacter gyllenbergii* (T)	100	AJ293694	*Proteobacteria/Acinetobacter*	Strictly aerobic, grows at 25–37°C, incapable of dissimilative denitrification, heterotrophic bacteria (Nemec et al., 2009).

In the first microbial identification (27th day), which corresponded to the low DO and high temperature MFC operating conditions (stage 2 in Figure 2), 12 bands were identified (Table 1). The results showed that the microbial community could be divided into 4 phyla, 4 classes and 8 genera. The phylum *Firmicutes* (bands 2, 3, 4, 5, 7, 8, 9, 10, and 13) was the predominant bacteria, while the phyla *Ignavibacteriae* (band 1), *Chloroflexi* (band 6) and *Proteobacteria* (band 14) were the subdominant groups.

Within the *Firmicutes* phylum, bands 2, 4, 5, 7, 8, and 9 belonged to the genus *Ureibacillus*, related to a species of *U. thermosphaericus* (similarity 91.2–100%), which was reported to be a thermophilic bacteria (37–60°C, optimum 50–60°C) with heterotrophic growth in aerobic environments and no ability for nitrate reduction (Fortina et al., 2001). This species was inferred to dominate the nitrification in the cathode of the MFC under low DO and high temperature conditions.

Bands 1 and 6 belonged to the genera *Ignavibacterium* and *Unclassified Anaerolineaceae*, respectively, which grow under anaerobic conditions, are heterotrophic and cannot utilize nitrate as electron acceptors (Yamada et al., 2006; Iino et al., 2010). These species were inferred to dominate the anaerobic degradation of COD.

Bands 3 (1) and 13 belonged to the genera *Geobacillus* and *Anoxybacillus*, respectively, which grow under aerobic conditions, are heterotrophic and can reduce nitrate (Poli et al., 2006; Inan et al., 2013). These species were inferred to dominate the aerobic denitrification.

Bands 3 (2) and 10 belonged to the genera *Anoxybacillus* and *Bacillus*, respectively, which grow under anaerobic conditions, are heterotrophic and can reduce nitrate (Kim et al., 1998; Cihan et al., 2014). These two species were inferred to be responsible for the anaerobic denitrification in stage 2 (Figure 2).

Band 14 belonged to the genus *Cucumibacter*, which grows under aerobic conditions, is heterotrophic and cannot utilize nitrate as electron acceptors (Hwang and Cho, 2008). This species was inferred to be responsible for the aerobic degradation of COD in stage 2 (Figure 2).

### Performance of the microbial community at high DO and high temperature

In the second microbial identification (83th day), which corresponded to the high DO and high temperature MFC operating conditions (stage 4 in Figure 2), 7 bands were identified (Table 2). The results showed that the microbial community could be divided into 1 phylum, 7 classes and 8 genera. The phylum *Proteobacteria* (bands 15, 18, 24, 25, 26, 27, and 28) was the predominant and unique phylum. This result agrees with other studies that indicated that *Proteobacteria* dominates some cathodic denitrifying biofilms (Wrighton et al., 2010).

In the phylum *Proteobacteria*, bands 15, 24 and 27 belonged to the genera *Aquamicrobium, Alishewanella*, and *Brachymonas* or *Comamonas*, respectively. These three species can grow under aerobic conditions, are heterotrophic, can utilize nitrate as electron acceptors (Hiraishi et al., 1995; Roh et al., 2009; Jin et al., 2013)and were inferred to dominate the aerobic denitrification under the high DO conditions of stage 4 (Figure 2). In particular, *Comamonas denitrificans* (band 27) has been reported to switch the metabolic pathway for extracellular electron transfer (Xing et al., 2010). A species in the *Comamonas* genus is known to be an aerobic denitrifier, and *C. denitrificans* is the only species in the *Comamonas* genus that can reduce nitrate to nitrogen gas and contains cd1-type nitrite reductase (Gumaelius et al., 2001). Therefore, we propose that *C. denitrificans* be considered a species of ADB. The other two species were inferred to belong to ADB.

Bands 18, 26 and 28 belonged to the genera *Pelomonas, Altererythrobacter* and *Acinetobacter*, respectively. These three species can grow under aerobic conditions, are heterotrophic, cannot utilize nitrate as electron acceptors (Xie and Yokota, 2005; Kwon et al., 2007; Nemec et al., 2009) and might have dominated the aerobic degradation of COD in stage 4 (Figure 2).

Band 25 belonged to the genus *Brevundimonas*, which grows under aerobic conditions and cannot reduce nitrate, and 90% of the strains are autotrophic (Segers et al., 1994). This species was inferred to be a nitrifier.

Based on this analysis, at high temperatures, the increase in DO resulted in a change in the predominant species from thermophilic autotrophic nitrifiers and facultative heterotrophic denitrifiers at low DO concentrations to thermophilic ADB at high DO concentrations during the operation of the MFC. Three species from the genera *Aquamicrobium, Brachymonas* or *Comamonas*, and *Alishewanella* were inferred to belong to ADB and dominate the aerobic denitrification at high levels of DO. Some aerobic denitrifiers were known to be heterotrophic nitrifiers, which might benefit SND under aerobic conditions. Therefore, autotrophic nitrifiers were replaced, and ADB evolved to be the predominant bacteria at high DO concentrations in stage 4 of the operation. This result is similar to the study by Feng et al., who indicted that aerobic denitrification in the cathode chamber is an important pathway for nitrite and nitrate removal (Feng et al., 2015).

### Mechanism of the cathode chamber

**Figure 7:**
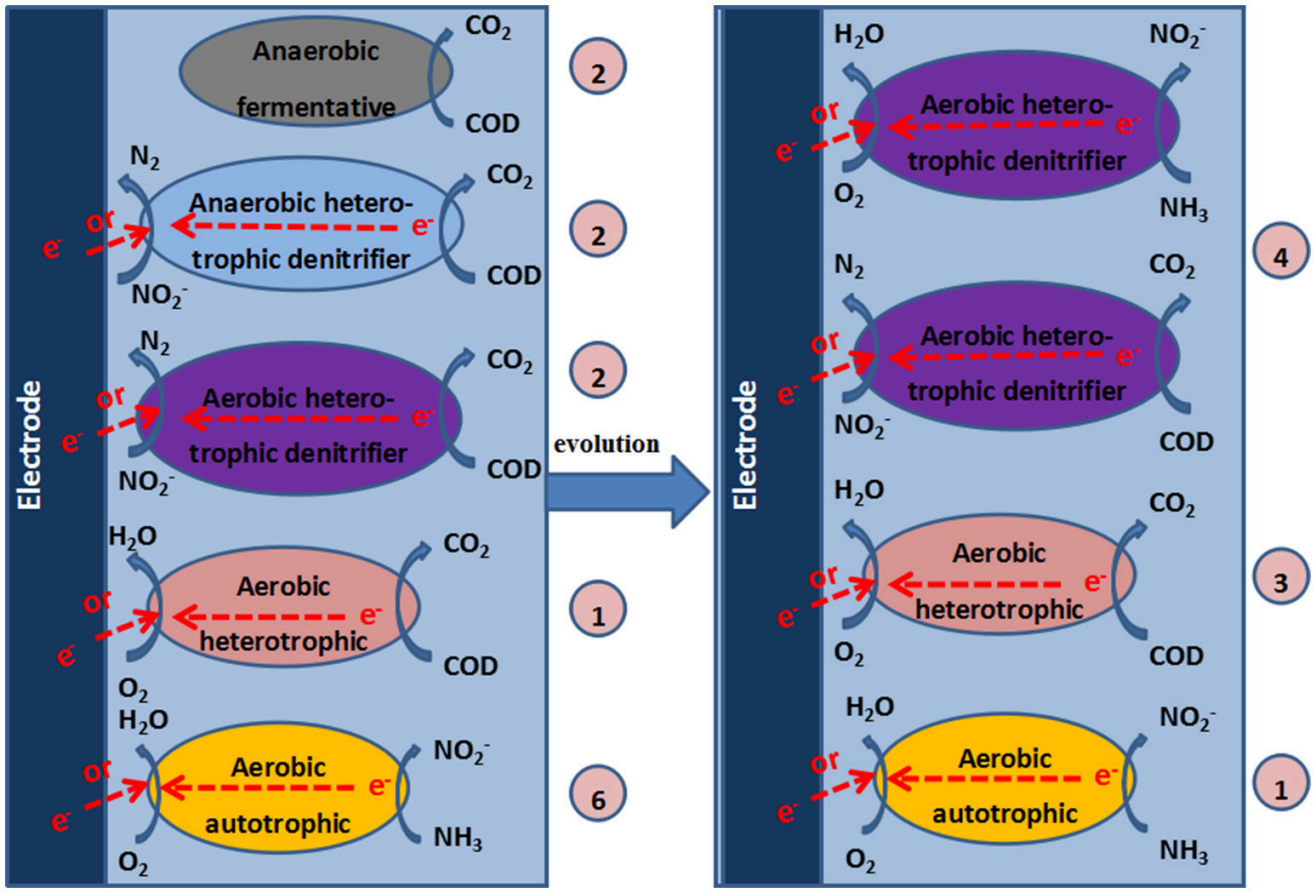
**Mechanisms in the cathode chamber of the MFC**. The numbers in the circles represent the quantities of DGGE bands that correspond to the identified bacteria.

Based on the analysis of the composition of the microbial community and the experimental results, we speculated on the possible reactions in the cathode of the MFC (Figure 7).

The mechanism of aerobic denitrification was determined by studying the aerobic denitrifier *T. pantotropha*. The cooperative breathing theory, which was proposed by Robertson et al. (1988), is widely recognized as the aerobic denitrification mechanism. Cooperative breathing theory means that both ${\text{NO}}_{3}^{-}$ and O_2_ can be used as the final electron receptors. Therefore, the denitrifiers can transfer electrons from the reduced substance to ${\text{NO}}_{3}^{-}$ or O_2_, and the denitrification can occur in an aerobic environment. According to the electron transport model proposed by Willson and Bouwer (1997), both ${\text{NO}}_{3}^{-}$ and O_2_ can be used as the final electron receptors, while the denitrifiers can transfer electrons from the reduced substance to O_2_ or ${\text{NO}}_{3}^{-}$ through nitrate reductase. Moreover, a carbon source is required for ADB. The higher the concentration of the carbon source is, the faster the aerobic denitrification rate will be (Robertson and Kuenen, 1983). Huang and Tseng (2001) indicated that the denitrification rate was highest when C/N was 5 and decreased with increasing C/N when C/N was greater than 5 and acetate was used as the carbon source. However, ADB did not appear to be required at such a high C/N ratio in our study. The influent C/N for the cathode chamber of the MFC was less than 0.3, possibly because the electrons provided by the electrode of the MFC were sufficient for the growth of aerobic bacteria. The aeration cathode chamber of the MFC may be beneficial for the growth of ADB.

Huang et al. indicated that DO was a key factor for aerobic denitrification (Xing et al., 2010). DO concentrations of 2–6 mg/L were beneficial for the growth of aerobic bacteria and the denitrification performance. As a result of the coexistence of aerobic respiration and the denitrifying reductase in one system, both O_2_ and ${\text{NO}}_{\text{x}}^{-}$ were indispensable for the growth of ADB and were used as electron acceptors. Therefore, at the high DO levels in this study (3.0–4.2 mg/L), the activity of ADB improved, and the denitrification performance of ADB was enhanced. The results that showed that the production of N_2_O and the TN removal efficiency were higher with a 50 Ω external resistance than with a 100 Ω resistance at high levels of DO demonstrated that electrode denitrification with ADB occurred in the cathode of the MFC.

## Conclusions

The increase of DO resulted in a change in the predominant species from thermophilic autotrophic nitrifiers and facultative heterotrophic denitrifiers at low DO levels to thermophilic ADB at high DO levels in the cathode of the MFC. The predominant phylum changed from *Firmicutes* to *Proteobacteria*, and the predominant class changed from *Bacilli* to *Alpha, Beta*, and *Gamma Proteobacteria*.

ADB is beneficial for achieving higher MFC voltages under high DO conditions, while traditional heterotrophic denitrification is conducive to higher TN removal under low DO conditions.

SND in the aeration cathode of the MFC may be beneficial for the growth of ADB.

## Author contributions

JZ conceived and designed the experiments, and JW and SW performed the experiments. JW analyzed the data and wrote the paper, and JZ, SW, XL, BH, and XD reviewed and edited the manuscript. All of the authors approved the manuscript to be published and agreed to be accountable for all aspects of the work and for questions related to the accuracy of the results.

### Conflict of interest statement

The authors declare that the research was conducted in the absence of any commercial or financial relationships that could be construed as a potential conflict of interest.
