# Supplementary material for: The Denitrification Characteristics and Microbial Community in the Cathode of an MFC with Aerobic Denitrification at High Temperatures
Source: Front Microbiol. 2017 Jan 19;8:9. doi: 10.3389/fmicb.2017.00009 (PMC5243800; doi:10.3389/fmicb.2017.00009)
Supplement: Supplementary file 1 [file DataSheet1.DOCX]

**Supplementary Material**

The denitrification characteristics and microbial community in the cathode of an MFC with aerobic denitrification at high temperatures

Jianqiang Zhao^1,2^, Jinna Wu^1^, Xiaoling Li^1^, Sha Wang^1^, Bo Hu^1^, Xiaoqian Ding^1^

^1^School of Environmental Science and Engineering, Chang'an University, Xi'an, China

^2^Key Laboratory of Subsurface Hydrology and Ecological Effect in Arid Region of Ministry of Education,

Jianqiang Zhao (Corresponding author)

Tel.:+86-029-13709188286; Fax: +86 029 82334566; E-mail: 626710287@qq.com

**The following are included as supporting information for this paper:**

Number of pages: 4

The sequences from DGGE bands are as flowing:

>Seq1

CCTACGGGAGGCAGCAGTAAGGAATATTGGTCAATGGGAGAAATCCTGAACCAGCAACGCCGCGTGAAGGACGAAGGGCTTTTAGCTTGTAAACATCTTTTTTGAGGGAAAAATATACGATTACTTCGTGTTGATTGTACCTTGAGAATAAGCCCCGGCTAACTACGTGCCAGCAGCCGCGGTAAT

>Seq2

CCTACGGGAGGCAGCAGTAGGGAATCTTCCACAATGGGCGAAAGCCTGATGGAGCAACGCCGCGTGGGCGAAGAAGGTCTTCGGATCGTAAAGCTCTGTTGTAAGGGAAGAATAAGTGCAGTAGTAACTGGCAGCACCTTGACGGTACCTTACTAGAAAGCCACGGCTAACTACGTGCCAGCAGCCGCGGTAAT

>Seq3

CCTACGGGAGGCAGCAGTAGGGAATCTTCCGCAATGGACGAAAGTCTGACGGAGCGACGCCGCGTGAGCGAAGAAGGTCTTCGGATCGTAAAGCTCTGTTGTTAGGGAAGAACAAGTACCGTTCGAATAGGGCGGTACCTTGACGGTACCTAACGAGAAAGCCACGGCTAACTACGTGCCAGCAGCCGCGGTAAT

>Seq4

CCTACGGGAGGCAGCAGTAGGGAATCTTCCACAATGGGCGAAAGCCTGATGGAGCAACGCCGCGTGAGCGAAGAAGGTCTTCGGATCGTAAAGCTCTGTTGTAAGGGAAGAACAAGTGCAGTAGTAACTGGCTGCACCTTGACGGTACCTTACTAGAAAGCCACGGCTAACTACGTGCCAGCAGCCGCGGTAAT

>Seq5

CCTACGGGAGGCAGCAGTAGGGAATCTTCCACAATGGGCGAAAGCCTGATGGAGCAACGCCGCGTGAGCGAAGAAGGTCTTCGGATCGTAAAGCTCTGTTGTAAGGGAAGAACAAGTGCAGTAGTAACTGGCTGCACCTTGACGGTACCTAACCAGAAAGCCACGGCTAACTACGTGCCAGCAGCCGCGGTAAT

>Seq6

CCTACGGGAGGCAGCAGTGAGGAATATTGCGCAATGGGCGAAAGCCTGACGCAGCGACGCCGCGTGGACGATGAAGGTCTTCGGATCGTAAAGTCCTTTTCTGCGTGACGAGGAAGGACGGTAGCGCAAGAATAAGTGTCGGCTAACTACGTGCCAGCAGCCGCGGTAAT

>Seq7

CCTACGGGAGGCAGCAGTAGGGAATCTTCCGTAATGGACGAAAGTCTGATGGAGCAACGCCGCGTGAGCGAAGAAGGTCTTCGGATCGTAAAGCTCTGTTGTAAGGGAAGAACAAGTGCAGTAGTAACTGGCTGCACCTTGACGGTACCTTACTAGAAAGCCACGGCTAACTACGTGCCAGCAGCCGCGGTAAT

>Seq8

CCTACGGGAGGCAGCAGTAGGGAATCTTCCACATTGGGCGAAAGCCTGATGGAGCAACGCCGCGTGAGCGAAGAAGGTCTTCGGATCGTAAAGCTCTGTTGTAAGGGAAGAACAAGTGCAGTAGTAACTGGCTGCACCTTGACGGTACCTTACTAGAAAGCCACGGCTAACTACGTGCCAGCAGCCGCGGTAAT

>Seq9

CCTACGGGAGGCAGCAGTGAGGAATCTTCCACAATGGGCGAAAGCCTGATGGAGCAACGCCGCGTGAGCGAAGAAGGTCTTCGGATCGTAAAGCTCTGTTGTAAGGGAAGAACAAGTGCAGTAGTAACTGGCTGCACCTTGACGGTACCTTACTAGAAAGCCACGGCTAACTACGTGCCAGCAGCCGCGGTAAT

>Seq10

CCTACGGGAGGCAGCAGTAGGGAATCTTCCGCAATGGACGAAAGTCTGACGGAGCAACGCCGCGTGAGCGAAGAAGGTCTTCGGATCGTAAAGCTCTGTTGTTAGGGAAGAACAAGTACCGTTCGAATAGGGCGGTACCGTGACGGTACCTAACCAGAAAGCCACGGCTAACTACGTGCCAGCAGCCGCGGTAAT

>Seq13

CCTACGGGAGGCAGCAGTAGGGAATCTTCCGCAATGGACGAAAGTCTGACGGAGCAACGCCGCGTGAGCGAAGAAGGCCTTCGGGTCGTAAAGCTCTGTTGTTAGGGAAGAACAGGTAACGTAGTAACTGGCGTTACTGTGACGGTACCTAACGAGAAAGCCACGGCTAACTACGTGCCAGCAGCCGCGGTAAT

>Seq14

CCTACGGGAGGCAGCAGTGGGGAATATTGGACAATGGGCGAAAGCCTGATCCAGCCATGCCGCGTGAGTGATGAAGGCCCTAGGGTTGTAAAGCTCTTTCAGTGGGGAAGATAATGACGGTACCCACAGAAGAAGCCCCGGCTAACTTCGTGCCAGCAGCCGCGGTAAT

>Seq15

CCTACGGGAGGCAGCAGTGGGGAATATTGGACAATGGGCGAAAGCCTGATCCAGCCATGCCGCGTGAGTGATGAAGGCCCTAGGGTTGTAAAGCTCTTTCACCGGGGAAGATAATGACGGTACCCGGAGAAGAAGCCCCGGCTAACTTCGTGCCAGCAGCCGCGGTAAT

>Seq18

CCTACGGGAGGCAGCAGTGGGGAATTTTGGACAATGGGCGCAAGCCTGATCCAGCCATGCCGCGTGCGGGAAGAAGGCCTTCGGGTTGTAAACCGCTTTTGTCAGGGAAGAAAAGGTTCTGGTTAATACCTGGGACTCATGACGGTACCTGAAGAATAAGCACCGGCTAACTACGTGCCAGCAGCCGCGGTAAT

>Seq24

CTACGGGAGGCAGCAGTGGGGAATATTGGACGATGGGCGCAAGCCTGATCCAGCCATGCCGCGTGTGTGAAGAAGGCCTTAGGGTTGTAAAGCACTTTCAGTGGGGAGGAAGGGTGTTGTGTTAATAGTACAGCACTTTGACGTTACCCACAGAAGAAGCACCGGCTAACTCCGTGCCAGCAGCCGCGGTAAT

>Seq25

CCTACGGGAGGCAGCAGTGGGGAATCTTGCGCAATGGGCGAAAGCCTGACGCAGCCATGCCGCGTGAATGATGAAGGTCTTAGGATTGTAAAATTCTTTCACCGGGGACGATGATGACGGTACCCGGAGAAGAAGCCCCGGCTAACTTCGTGCCAGCAGCCGCGGTAAT

>Seq26

CCTACGGGAGGCAGCAGTGGGGAATATTGGACAATGGGCGAAAGCCTGATCCAGCAATGCCGCGTGAGTGATGAAGGCCCTAGGGTTGTAAAGCTCTTTTACCCGGGATGATAATGACAGTACCGGGAGAATAAGCACCGGCTAACTCCGTGCCAGCAGCCGCGGTAAT

>Seq27

CCTACGGGAGGCAGCAGTGGGGAGTTTTGGACAATGGGCGCAAGCCTGATCCAGCAATGCCGCGTGCAGGATGAAGGCCTTCGGGTTGTAAACTGCTTTTGTACAGAACGAAAAGTCTTGGGTTAATACCCTGGGACCATGACGGTACTGTAAGAATAAGCACCGGCTAACTACGTGCCAGCAGCCGCGGTAAT

>Seq28

CCTACGGGAGGCAGCAGTGGGGAATATTGGACAATGGGCGGAAGCCTGATCCAGCCATGCCGCGTGTGTGAAGAAGGCCTTTTGGTTGTAAAGCACTTTAAGCGAGGAGGAGGCTACTAGTACTAATACTACTGGATAGTGGACGTTACTCGCAGAATAAGCACCGGCTAACTCTGTGCCAGCAGCCGCGGTAAT
